# Evaluation of techniques for diagnosis of *Trypanosoma vivax* infections in naturally infected cattle in the Zona da Mata Mineira

**DOI:** 10.1590/S1984-29612022007

**Published:** 2022-02-25

**Authors:** Jefferson Filgueira Alcindo, Maria Clara Guimarães Vieira, Thamiris Vilela Pereira Rocha, Cinthya Brillante Cardinot, Maurício Deschk, Gláucia Guimarães Amaral, Rafael Ferreira de Araujo, Carina Franciscato, Kayo José Garcia de Almeida Castilho, Rosângela Zacarias Machado, Marcos Rogério André

**Affiliations:** 1 Laboratório de Patologia Clínica, Departamento de Medicina Veterinária, Universidade Federal de Juiz de Fora – UFJF, Juiz de Fora, MG, Brasil; 2 Laboratório de Imunoparasitologia, Departamento de Patologia, Reprodução e Saúde Única, Faculdade de Ciências Agrárias e Veterinárias – FCAV, Universidade Estadual Paulista – UNESP, Jaboticabal, SP, Brasil

**Keywords:** Trypanosomiasis, dairy cattle, diagnosis, laboratory techniques, Tripanossomíase, bovinos leiteiros, diagnóstico, técnicas laboratoriais

## Abstract

This study aimed to evaluate diagnostic techniques for trypanosomiasis, caused by *Trypanosoma vivax*, in naturally infected cattle in Minas Gerais, Zona da Mata. The deaths of six lactating cows with similar clinical conditions—characterized by hyporexia, hypogalactia, and recumbency—had been reported from one property. Initially, two animals were examined and diagnosed with trypanosomiasis through identification of the protozoan in a blood smear. After the initial diagnosis, all lactating cows (n=37) on the property were examined, and blood samples were collected for tests including whole blood smear, buffy coat smear, Woo's technique, enzyme-linked immunosorbent assay (ELISA), and polymerase chain reaction (PCR). Woo's test, buffy coat smears, and whole blood smears indicated that 4/37 (10.81%) animals were positive for trypanosomiasis, whereas ELISA and PCR indicated that 33/37 (89.19%) and 27/37 (72.97%) animals, respectively, were positive. The agreement obtained between parasitological techniques was classified as high, while between ELISA and PCR, no agreement. In conclusion, parasitological techniques have a low capacity to identify infected animals in the chronic stage of *T. vivax* infection. Therefore, techniques such as PCR and/or ELISA should be used to minimize the occurrence of false negatives.

## Resumo

Este estudo objetiva avaliar as técnicas de diagnóstico da tripanossomíase, causada pelo *Trypanosoma vivax*, em bovinos naturalmente infectados, em Minas Gerais, Zona da Mata. A morte de seis vacas em lactação com condições clínicas semelhantes - caracterizadas por hiporexia, hipogalaxia e decúbito - foi relatada em uma propriedade. Inicialmente, dois animais foram examinados e diagnosticados com tripanossomíase através da identificação do protozoário em esfregaço sanguíneo. Após o diagnóstico inicial, todas as vacas em lactação (n = 37) na propriedade foram examinadas, e amostras de sangue foram coletadas para testes, incluindo esfregaço de sangue total, esfregaço de capa leucocitária, técnica de Woo, ensaio imunoenzimático (ELISA) e reação em cadeia da polimerase (PCR). O teste de Woo, os esfregaços de capa leucocitária e de sangue total indicaram que 4/37 (10,81%) animais foram positivos para tripanossomíase, enquanto ELISA e PCR indicaram que 33/37 (89,19%) e 27/37 (72,97%) animais, respectivamente, foram positivos. A concordância entre técnicas parasitológicas foi classificada como alta, enquanto entre ELISA e PCR, sem concordância. As técnicas parasitológicas apresentam baixa capacidade para identificar animais infectados na fase crônica da infecção por *T. vivax*. Dessa forma, técnicas como PCR e/ou ELISA devem ser utilizadas para minimizar a ocorrência de falsos negativos.

## Introduction

The trypanosomiases are cosmopolitan diseases caused by protozoa of the *Trypanosoma* genus, with *Trypanosoma vivax* being the agent most relevant to etiology and pathogenicity in South American cattle (Jones & Dávila, 2001; Batista et al., 2008).

In Minas Gerais, the first report of *T. vivax* was in the municipality of Igarapé in 2007 (Carvalho et al., 2008). However, the disease has spread to other regions of the state (Cuglovici et al., 2010; Meneses, 2016; Reis et al., 2019). In one study, the estimated seroprevalence for the state was 2.38% and positive serological results were found in all mesoregions, including the Zona da Mata Mineira, with 1.67% of seroprevalence.

Despite the low seroprevalence, the parasite is dispersed in Minas Gerais State, which makes seronegative herds even more susceptible to the disease (Meneses, 2016). Other outbreaks of *T. vivax* in states bordering Minas Gerais, including São Paulo (Cadioli et al., 2012), Rio de Janeiro (Costa et al., 2020), Goiás (Bastos et al., 2020) and Bahia (Gomes et al., 2021) were also reported.

The main risk factors in the transmission of the agent in dairy cattle herds from Minas Gerais already reported involving the acquisition of animals without prior knowledge of their health status (Meneses, 2016), the presence of a population of hematophagous flies acting as mechanical vectors (Cuglovici et al., 2010) and needle sharing between animals, especially for the application of oxytocin (Reis et al., 2019).

The clinical signs observed in animals infected with *T. vivax* are nonspecific and similar to those observed in other parasitic diseases, such as babesiosis and anaplasmosis. Therefore, diagnosis is difficult and must be performed using laboratory tests. The decision to use parasitological, serological, and/or molecular techniques will depend on the stage of the disease and consequently on the variations between the specificity and sensitivity of the tests (Meneses, 2016). Parasitological techniques such blood smear and Woo’s test are easy to perform (Osório et al., 2008) and promote the identification of infected animals in the acute phase of the disease (Bastos et al., 2020). On the other hand, serological tests, such as the ELISA, which is used to detect antibodies against *T. vivax*, are considered to be more sensitive. Furthermore, molecular detection methods such as PCR have presented good results, as it is possible to determine the trypanosome species under investigation (Dagnachew & Bezie, 2015). This study aimed to evaluate diagnostic techniques for *T. vivax* in naturally infected cattle in the Zona da Mata region of Minas Gerais, as well as to describe the epidemiological aspects of the disease in the region under study.

## Materials and Methods

### Farm characterization, animals and clinical signs

The study was performed in January 2019, on a dairy property located in the municipality of Cipotânea, Zona da Mata region, Minas Gerais, at latitude 20°54′23″ S and longitude 3°21ʹ37″ W, with an area of ​​approximately 39 ha. The herd consisted of 37 lactating Holstein cows, aged 4–6 y, raised in a semi-intensive system. The animal owner reported the death of six lactating cows that presented a clinical condition characterized by hyporexia, hypogalactia, and decubitus.

The duration, of the clinical picture of the cows that died, was variable, from three to fifteen days. In addition to the mentioned signs, the animals' tutor reported that one of the animals presented blindness and corneal opacity. From the first clinical case until the moment the two cows were evaluated, 90 days had passed. All animals that showed clinical signs were treated under the guidance of a veterinarian, however, the medication used was not specific for the parasite.

Regarding the management of the property, after weaning, all the calves were sold and the replacement of cows was made with the purchase of heifers. The acquired cows had a varied origin, most of them from the state of Minas Gerais, and were exempt from quarantine. The last batch purchased had been introduced on the farm 60 days before the onset of the problem and consisted of 15 animals from the municipality of Lamim-MG, also located in the Zona da Mata Mineira. The same problem has not been reported in the close farms.

It was also common practice to use oxytocin to stimulate the ejection of milk at the time of milking, and all animals received the medication in both milkings. The needles used for the applications were shared between the animals and the handling was not supervised by a veterinarian. In addition, during visits to the property, a large number of stable flies (*Stomoxys calcitrans*) were observed, causing the animals to fleece.

During the first visit, two lactating cows showing chronic clinical signs were examined and diagnosed with trypanosomiasis based on the identification of *T. vivax* in a blood smear. After the two animals were diagnosed, all 37 lactating cows on the property were examined; blood samples—with and without anticoagulant—were collected for further laboratory tests, namely buffy coat, whole blood smear, Woo's test, ELISA, and PCR.

### Diagnostic methods

The whole blood and white blood cell smear slides were stained by the rapid panoptic method and examined under an immersion optical microscope (100 × magnification). Woo's test was performed by centrifuging whole blood with anticoagulant in microhematocrit tubes and identifying trypanosomes by direct observation of the capillary under a microscope at 40× magnification (Woo, 1970).

DNA extraction from whole blood (with EDTA) was performed based on the genomic DNA isolation protocol described by Kuramae-Izioka (1997). All samples were subjected to PCR for the endogenous *Glyceraldehyde-3-phosphate dehydrogenase* (*GAPDH*) gene, as described by Birkenheuer et al. (2003), using oligonucleotide primers (GAPDH F 5ʹ-CCTTCATTGACCTCAACTACAT-3ʹ and GAPDH R 5ʹ-CCAAAGTTGTCATGGATGACC-3ʹ; IDT®, Coralville, USA). For the diagnosis of *T. vivax* by PCR, the technique described by Cortez et al. (2009) was used, employing oligonucleotide primers (TviCatL 5ʹ-GCCATCGCCAAGTACCTCGCCGA-3ʹ and DT0155 5ʹ-TTAAAGCTTCCACGAGTTCTTGATGATCCAGTA-3ʹ; IDT®, USA) flanking a 177 bp fragment of the *cathepsin L* (*CatL*)*-like* catalytic domain region gene, which encodes the enzyme CatL-like in *T. vivax*. Reactions were conducted in a T100 Thermal Cycler (Bio-Rad, USA), accompanied by DNA from the “Lins” isolate of *T. vivax* (Cadioli et al., 2012) as a positive control, and by autoclaved Ultra Pure DNAse/RNAse-free distilled water (Invitrogen®) as a negative control.

PCR assays targeting the amplification of fragments of endogenous gene *GAPDH* from host and *CatL-like* genes from*T. vivax*were carried out in a thermocycler (T100 Thermal Cycler, Bio-Rad). The amplified products were submitted to horizontal electrophoresis in 1.0% agarose gel for the *GAPDH* gene and 2.0% for the *CatL-like* gene, stained with ethidium bromide in TEB buffer of pH 8.0 (44.58 M Tris-base; 0.44 M boric acid; 12.49 mM EDTA). The results were visualized and analyzed using an ultraviolet light transilluminator coupled to a computer image analysis program (ChemiDoc Imaging System, Bio-Rad).

ELISA was performed according to the method described by Machado et al. (1997) and Aquino et al. (1999), with minor modifications. Flat-bottomed Nunc MaxiSorp plates (Thermo Fisher Scientific, Massachusetts, USA) were sensitized with 0.1 µg/mL of diluted soluble “TvY protein” (recombinant *T. vivax* antigen), which was cloned and expressed by Imunodot Diagnostics and was in the final stages of registration at the Brazilian Ministry of Agriculture, Livestock, and Supply. Control sera and those to be tested were diluted to 1:100. The conjugate used was rabbit IgG anti-bovine IgG coupled to alkaline phosphatase (A0705, Sigma-Aldrich,) at 1:30,000 dilution. All samples were tested in duplicates.

To determine the cut-off point of the plates, two negative controls and two positive controls were used. Negative controls were from two adult cattle from a herd located in a region where trypanosomiasis was not endemic, and which had been previously tested using molecular tests. Negative results for *T. vivax* infection were obtained for all tests. Positive controls were from two adult cattle experimentally infected with the “Lins” isolate of *T. vivax* (Fidelis et al., 2016). The mean and standard deviation (SD) of the optical densities (OD) obtained from the negative controls of all plates were calculated, and from these results the cutoff was established according to the following equation, described by Gomes et al. (2008):


Cutoff = Mean of negative controls x 2.5 (1)

### Statistical analysis

Statistical analysis, specifically the kappa test, of the comparison between the tests for the diagnosis of natural infection by *Trypanosoma vivax* was performed using SAS^®^ (SAS, 2004), establishing the index of agreement between the techniques. Agreement was classified according to Landis & Koch (1977): kappa index values < 0 = *no agreement*; values 0–0.19 = *low agreement*; values 0.20–0.39 = *regular agreement*; values 0.40–0.59 = *moderate agreement*; values 0.60–0.79 = *substantial agreement*; and values 0.80–1.00 = *high agreement*.

## Results and Discussion

The first two cows of the herd of 37 had been initially diagnosed with trypanosomiasis by identification of trypomastigote forms of protozoans in a blood smear, which are morphological characteristics compatible with *T. vivax* (Dagnachew & Bezie, 2015). The diagnosis of the disease had already been reported in the region (Meneses, 2016) and some risk factors may have contributed to the introduction of the agent in the property, such as misuse of needles and syringes during the application of oxytocin, the free transit of animals, and the presence of hematophagous flies.

The shared use of needles and syringes, especially during the application of oxytocin before milking, is an important risk factor for the occurrence of the disease (Batista et al., 2008; Bastos et al., 2020). The entry of animals from herds from other regions without prior quarantine should be considered in the epidemiology of the outbreak (Costa et al., 2020). It is known that the free transit of cattle is a potential risk for the occurrence of trypanosomosis outbreaks (Pimentel et al., 2012). Studies have reported a possible relationship between the presence of the stable fly and the occurrence of the disease (Cuglovici et al., 2010; Batista et al., 2008), although Bastos et al. (2020) not having considered the presence of arthropods as a relevant risk factor in the occurrence of *T. vivax*.

Of the parasitological techniques used, 10.81% (4/37) of the animals were positive for trypanosomiasis (Table 1). The results of parasitological tests were not similar to that observed in experimentally infected cattle, where Woo's technique proved to be the best method, with a detection rate of 44.4% (Fidelis et al., 2019).

**Table 1:** Results of diagnostic tests for *Trypanosoma vivax* performed in cattle herd of the naturally infected in Zona da Mata region of Minas Gerais.

**Test**	**Results**	
	**Positive**	**Negative**
Woo’s technique	4/37 (10.81%)	33/37 (89.19%)
Buffy coat smear	4/37 (10.81%)	33/37 (89.19%)
Whole Blood smear	4/37 (10.81%)	33/37 (89.19%)
ELISA	33/37 (89.19%)	4/37 (10.81%)
PCR	27/37 (72.97%)	10/37 (27.03%)

Although the parasitological techniques presented low sensitivity (Fidelis et al., 2019; Costa et al., 2020), in this work such results were relevant, showing a high parasite load in the sampled animals. The high parasitemia in these cases favors the transmission of *T. vivax* by mechanical vectors (Osório et al., 2008) and possibly by iatrogenic transmission, through the sharing of needles and syringes during oxytocin applications.

Using the results of ELISA, we obtained a Cutoff of 0.293 by the mathematical formula described above, and the mean ± SD of the negative and positive controls was 0.117 ± 0.003 and 1,061 ± 0,004, respectively. The mean absorbance value obtained from positive and negative animals was 0.688 and 0.194, respectively. Serological testing indicated that 89.19% (33/37) of the animals had anti-*T. vivax* antibodies, indicating exposure to the parasite, though not necessarily current active infection (Cadioli et al., 2012; Fidelis et al., 2019). Similar results found by Cadioli et al. (2012) showed a seroprevalence of 98.36%. Serological tests such as ELISA can detect anti-*T. vivax* antibodies circulating for long periods in untreated animals, including animals treated with non-specific anti-*Trypanosoma* drugs (Fidelis et al., 2019). Thus, it constitutes an important tool to assess the epidemiological status of the herd (Osório et al., 2008; Cuglovici et al., 2010), although it is not the most suitable for differentiating individuals with active infection from those undergoing treatment or spontaneously cured (Cadioli et al., 2012; Castilho et al., 2021). In addition, this serological technique has limitations for identifying antibodies during the first days of infection (Fidelis et al., 2019). These results highlight the importance of using high-sensitivity techniques such as ELISA in naturally infected animals to avoid further spreading of the parasitic infection due to a lack of effective diagnostic methods (Castilho et al., 2021).

**Figure 1:**
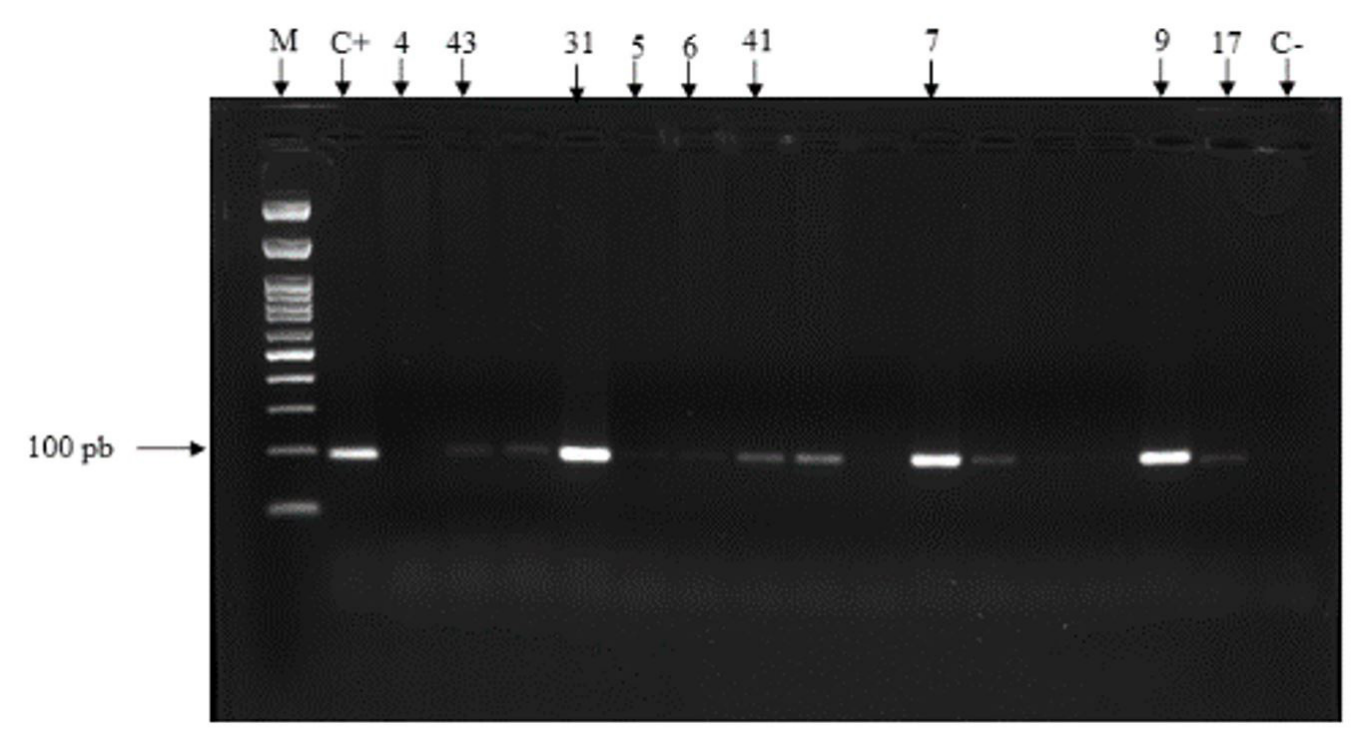
Electrophoresis in 2% agarose gel and cordoned with Ethidium bromide, illustrating the amplified PCR fragments of the CatL-Like gene of *Trypanosoma vivax*. C+ range: positive control; C- range: negative control; Samples at position 43, 31, 5, 6, 41, 7, 9 and 17: positive samples for *T. vivax*; Sample at position 4: negative sample for *T. vivax*; Sample at position M: molecular marker.

In the *T. vivax*-specific PCR assay, 72.97% (27/37) of the animals tested positive (Figure 1). In an outbreak reported in the state of Sergipe, 80% of animals were identified as having the disease through molecular testing (Vieira et al., 2017). PCR has high sensitivity and specificity in identifying positive individuals (Fidelis et al., 2019). Data indicate that the PCR-CatL technique can detect *T. vivax* DNA in periods of low parasitemia, including in aparasitemic moments (Cortez et al., 2009; Bastos et al., 2020), being, therefore, capable of detecting positive animals that are in the chronic phase of the disease. The association of parasitological and molecular techniques is effective in increasing the sensitivity for diagnosing the disease and decreasing false negatives in some studies, in which high rates of trypanosome infection were detected by molecular testing even in clinically healthy individuals who obtained results negative during parasitological tests, that is, with no detectable parasitemia (Pimentel et al., 2012; Garcia Pérez et al., 2020).

The results obtained using the Woo technique, buffy coat smear, and whole blood smear were in complete agreement, yielding a *kappa* index of 1.00 (Table 2). In naturally infected animals, the kappa index found, of 0.67, for Woo technique and a whole blood smear was considered substantial (Fidelis et al., 2019). However, ELISA and PCR did not reach an agreement, presenting a kappa index of -0.0137. This result is in agreement with that found by Castilho et al. (2021), who pointed out a kappa index of -0.02 when comparing the ELISA and PCR diagnostic tools. These findings are because the techniques in question are based on different assumptions and detection targets, reiterating the need to associate serological and molecular tools for the diagnosis of *T. vivax* in naturally infected herds (Castilho et al., 2021).

**Table 2:** Kappa index obtained through comparative analysis of whole blood smear, buffy coat smear, Woo technique, PCR and ELISA in cattle naturally infected by T. vivax in Zona da Mata, Minas Gerais.

	**Buffy coat**	**Woo’s technique**	**ELISA**	**PCR**
Whole blood smear	k *=* 1.000	k *=* 1.000	k *=* 0.0290	k *=* 0.0859
	C.I.( k; 95%):	C.I.( k; 95%):	C.I.( k; 95%):	C.I.( k; 95%):
	[1.000 ; 1.000 ]	[1.000 ; 1.000 ]	[-0.0103 ; 0.0682 ]	[-0.0073; 0.1791 ]
Buffy coat smear		k *=* 1.000	k *=*0.0290	k *=*0.0859
		C.I.( k; 95%):	C.I.( k; 95%):	C.I.( k; 95%):
		[1.000 ; 1.000 ]	[-0.0103 ; 0.0682 ]	[-0.0073 ; 0.1791 ]
Woo’s technique			k *=* 0.0290	k *=*0.0859
			C.I.( k; 95%):	C.I.( k; 95%):
			[-0.0103 ; 0.0682]	[-0.0073 ; 0.1791 ]
ELISA				k *=* -0.0137
				C.I.( k; 95%):
				[-0.2855 ; 0.2581]

k (Kappa index);

C.I (Confidence interval).

## Conclusions

For animals in the chronic stage of infection by *T. vivax,* parasitological techniques have a low capacity to identify infected animals, and techniques such as PCR and/or ELISA should be used to minimize the occurrence of false negatives. It is likely that the disease is disseminated in the study region and that risk factors such as frequent transit of animals, sharing of needles and failure to control stable flies played a role in the occurrence of the disease.
